# Correlation between glycemic control and quality of life of adolescents aged 13-18 years with type 1 diabetes mellitus

**DOI:** 10.1186/1687-9856-2013-S1-P30

**Published:** 2013-10-03

**Authors:** Elina Waiman, Bambang Tridjaja, Bambang Supriyatno

**Affiliations:** 1 Department of Child Health, Medical School, University of Indonesia, CiptoMangunkusumo Hospital, Jakarta, Indonesia

## 

The efforts to achieve good glycemic control might damage the quality of life (QoL). This issue has not been elaborated in Indonesia because there was no diabetes-specific quesionnaire available in Indonesian language previously. This study would like to find out whether there is a correlation between glycemic control and QoL of adolescents aged 13-18 years with type 1 diabetes mellitus (T1DM).

Cross-sectional study was held on patients who came to The Pediatric Endocrinology Clinic of Cipto Mangunkusumo Hospital or who joinned The Endocrinology Working Group Unit of Indonesian Pediatric Society programs during October to December 2010. Glycemic control was measured by hemoglobin A1c (HbA1c) levels. The audit of diabetes dependent quality of life-teen (ADDQoL-Teen) Indonesian questionnaire was used to assess the QoL.

A total of 36 adolescents participated in the study. Only seven subjects had a normal HbA1c level. Median HbA1c level was 8.7 (range, 5.6 to 15.1)%. Eighteen subjects considered their QoL as good or brilliant, while 14 subjects felt that diabetes had a negative impact. The most severe negative impact was felt on the matter of dietary aspect, but could be managed better by subjects with intensive treatment. There was no correlation between HbA1c level on the last examination and average weighted impact (AWI) score (rho = -0.15; p = 0.383) or between mean HbA1c level within 6 months and AWI score (rho = -0.15; p = 0.369).

This study did not show any correlation between glycemic control and QoL of adolescents with T1DM.

